# Sex Differences in the Impact of Exercise Volume on Subclinical Coronary Atherosclerosis: A Meta-Analysis

**DOI:** 10.1016/j.jacadv.2025.102251

**Published:** 2025-10-21

**Authors:** James M. Brophy

**Affiliations:** a McGill University Health Center, Centre for Health Outcomes Research (CORE), Montréal, Quebec, Canada; b McGill University, Montréal, Quebec, Canada

Abdelaziz et al.^1^ conclude “Males with high-volume exercise training (>3,000 metabolic equivalent of task-min/wk) exhibited a higher burden of calcified plaque by coronary artery calcium (CAC) score than male nonathletes, while no such difference was observed in female athletes.” They state the protocol was preregistered (CRD42024573617) but I was unable to find a mention of preplanned analyses according to sex. Of course, results from post hoc analyses carry less inferential validity. What was the prior probability that women would behave differently than men or that heavy exercising men would behave worse than moderately exercising men?

A more limited question is does this data show as stated by the authors that male athletes engaging in high-volume exercise compared to nonathletes have higher CAC scores than athletes with moderate volume exercise compared to nonathletes. The CAC values for male athletes compared to nonathletes was 1.2 (95% CI: −24.66 to 27.05; *P* = 0.9) for >3,000 group and 31.62 (95% CI: 10.66-52.58; *P* = 0.003) for group <3,000. Are these differences statistically significant? The answer is no. The risk difference for the interaction is 30.42, (95% CI: −2.86 to 63.70; *P* = 0.07). The key insight being “in making a comparison between 2 group, one should look at the statistical significance of the difference rather than the difference between their significance levels”.^2^

An even more important issue is the raw data. Male controls in the low-intensity group had means of 11.9, 143.3, and 189.07 and SDs of 26.1, 496.6, and 575.59, respectively. These SDs are massive relative to the means suggesting highly skewed distributions (the same phenomena observed in the high intensity group). Calculating mean differences assumes a normal distribution which appears untenable here as this would imply many negative CAC scores which is impossible.

Deviations from the preregistered protocol, questionable statistical assumptions and analyses weaken any possible inferences from this study.
